# Running demands during top-up conditioning sessions compared to competitive matches in elite Portuguese soccer players

**DOI:** 10.5114/biolsport.2025.151650

**Published:** 2025-06-24

**Authors:** João Ribeiro, Petrus Gantois, Fabiano de Souza Fonseca, Luis Suarez-Arrones, João Viana, Fábio Yuzo Nakamura

**Affiliations:** 1 Research Center in Sports Sciences, Health Sciences and Human Development (CIDESD), University of Maia, Maia, Portugal; 2 Department of Performance Optimization, GOD, Sporting Clube de Braga SAD, Braga, Portugal; 3 SC Braga Education, Braga, Portugal; 4 Associate Graduate Program in Physical Education UPE/UFPB, João Pessoa, Brazil; 5 Department of Optimization, Health, and Performance, DOSP, Sporting Clube de Braga, Braga, Portugal; 6 Federal Rural University of Pernambuco (UFRPE), Recife-PE, Brazil; 7 Department of Sports and Computer Science, Section of Physical Education and Sports, Universidad Pablo de Olavide, Seville, Spain

**Keywords:** External load, Compensatory training, Match-Day, Football, Substitutes

## Abstract

Soccer players who are non-starters typically experience reduced match loads, which can hinder their physical fitness and match readiness over time. This study aimed to investigate the running demands accumulated during top-up sessions in comparison to soccer matches. Twenty-six outfield soccer players from an elite Portuguese soccer team participated in this study. The following running variables were recorded: total distance (TD), running (14.4 to 19.7 km · h^−1^), high-speed running (HSR: 19.8 to 25.1 km · h^−1^), sprinting (≥ 25.2 km · h^−1^) distances, number of sprints (counts), number of accelerations (ACC; 2–3 m · s^−2^ and > 3 m · s^−2^), decelerations (DEC; 2–3 m · s^−2^ and > 3 m · s^−2^), and player load. A linear mixed-effects model was used to examine differences between top-up sessions and matches. Overall, non-starters accumulated lower running load during top-up sessions than matches for most of the variables analyzed, but in particular TD (p < 0.001; ES range 2.46 to 3.74), running (p < 0.001; ES range 2.93 to 3.90), HSR (p < 0.001; ES range 2.21 to 3.47), sprints events (p < 0.001; ES range 1.16 to 2.76), ACC > 3 m · s^−2^ (p < 0.005; ES range 0.98 to 1.37), DEC > 3 m · s^−2^ (p < 0.001; ES range 1.91 to 2.66), and player load (p < 0.001; ES range 2.34 to 3.23). Therefore, non-starters accumulated less than half of the total match distance for most of the running metrics during top-up sessions. These findings suggest that compensatory training should be designed to more closely replicate match demands, with particular attention to high-intensity demands, such as HSR and sprints.

## INTRODUCTION

During the in-season microcycle, matches represent the most physically demanding events of the week [1–3]. On average, the total distance covered by elite soccer players during a match ranges from 10 to 13 km [4–6], whereas the mean distance covered in running (> 14.4 km · h^−1^), high-speed running (HSR; 19.8–25.2 km · h^−1^), and sprinting (> 25.2 km · h^−1^) are reported to vary from 1348 to 3138 m, 482 to 1214 m, and 152 to 382 m, respectively [5, 7]. This physical loading accounts for 40–48% of the total weekly running distance covered during a typical microcycle, which includes one match and four training sessions [3]. In congested microcycles (i.e., more than one match per week), full matches may contribute up to 80–95% of HSR [8, 9]. Furthermore, over the course of the season, starters’ cumulative training distance accounts for only ~39%, ~31%, and ~9% of their respective season-long match-play running, HSR, and sprinting distances [1]. Collectively, these data suggest that participation in soccer match-play provides the most meaningful physical stimulus during in-season microcycles by enabling players to accumulate high-intensity activities, which are associated with physiological adaptations [10].

Soccer team squads consist of 22–26 players, but official matches only allow the participation of a limited number of players (i.e., 10 outfield starters and 3–5 substitutes) [11]. Notably, most of the substitutions during soccer matches occur at halftime or towards the end of the second half (i.e., 60–85 min) [12]. Therefore, a considerable number of players in the squad are not exposed to the total matchplay load during in-season microcycles (≤ 45 min). For example, during a typical week with one match, non-starters accumulated significantly lower weekly running (-31%) and HSR (-29%) distances than starters [3]. In addition, non-starters covered less than a full match distance in running (90%) and HSR (50%) during a congested microcycle [3]. Accumulating insufficient playing time and highspeed runs during matches may potentially drive some detraining effects [13, 14]. Likewise, under-loading non-starters in relation to maximal speed sprints may increase their risk of injury when they are suddenly exposed to very high-intensity activities [15, 16]. In fact, sprint training may be regarded as leading to positive changes in muscle architecture, such as increased biceps femoris long head fascicle length [17]. This is significant because shorter fascicle length has been identified as a modifiable risk factor for hamstring injuries in elite soccer players [18]. Therefore, conditioning training practices that overload non-starters during in-season microcycles are practically relevant to maintain their fitness levels, readiness to play, avoid detraining, and decrease the likelihood of injury risk.

To be physically prepared for the match, non-starters undergo compensatory training sessions (i.e., top-up sessions), typically on the match day or the following day (MD+1), in an attempt to replicate some of the match-play loads during the in-season microcycle [19, 20]. A recent survey on top-up conditioning practices reported that most soccer coaches (96%) implement extra top-up sessions for non-starters to compensate for their lack of match playing time and high-intensity activities [20]. For this purpose, several training modes (i.e., nonspecific running drills and/or small-sided games) have been implemented [19–21], with high-speed running and sprints representing the primary training goal of the top-up conditioning practices [20, 22]. However, a deeper understanding of match running performance dynamics is critical, particularly the role of seasonal variations in some metrics across different in-season phases [23]. Recent research in Portuguese professional soccer has demonstrated that these factors are significantly influenced by seasonal phases, with total distance and average speed emerging as the main determinants of match performance [23]. While small-sided games (SSG) in top-up sessions can replicate up to 80–86% of accelerations and decelerations, they may not sufficiently compensate for the high-speed running and sprinting demands (20–30% of match load), as observed in a Spanish professional club [21]. These findings emphasize the need to refine top-up practices by accounting for the specific match-running demands and seasonal trends observed in professional soccer across various leagues.

The use of global positioning system (GPS) technology allows soccer coaches to quantify running demands during training sessions relative to match events [3, 21, 24]. The quantification of running load during soccer matches enables scheduling conditioning practices for the soccer team squad [1, 25] based on individual needs. While extensive research has been conducted to better understand the matchrelated running demands in soccer players [2, 5, 6], there are few data examining the loading patterns during top-up sessions from elite soccer teams [3, 19–21]. Of note, playing position-specific trends exist regarding the individual match-playing time and physical load accumulated during competitive matches [12]. Thus, it is particularly important to explore these patterns within elite-level contexts to ensure relevance to high-performance teams. Such data may provide valuable insights to coaching staff for programming position-specific top-up conditioning practices for non-starters, helping to maintain the overall squad’s physical fitness and avoid undesirable spikes in physical loads across competitive microcycles [15, 16].

Therefore, more empirical data on compensatory loading strategies adopted by elite-level soccer teams for non-starters would be of interest in providing a practical framework for soccer coaching staff when planning top-ups. Accordingly, this study aimed to compare the accumulated running load at different intensities during postmatch top-up sessions and competitive matches in non-starters, considering playing positions.

## MATERIALS AND METHODS

### Study design

This is an observational and retrospective study that evaluated locomotor activities during matches and post-match top-up conditioning sessions in elite-level soccer players over the course of a full competitive season. Data were collected over 40 weeks, from August to March, while the team competed in four official competitions, including one international (UEFA Europa League).

### Sample selection

A total of 4,039 observations were recorded over the full season, comprising 753 observations during matches (n = 65) and 3,286 observations during training sessions (n = 180). Players were included in the analysis only if they had participated in at least one match (> 45 minutes) and one top-up session. Goalkeepers were not included in the data analysis due to the distinct nature of their physical demands and training.

In total, data were collected from 65 matches and their associated top-up sessions. The dataset consisted of 454 matches (median of 18 observations per player; range: 1 to 39) and 439 top-up conditioning sessions (median of 14 observations per player; range: 1 to 45). Of the 439 top-ups, 116 observations were made with central defenders (CD), 101 with fullbacks (FB), 82 with wide midfielders (WM), 102 with central midfielders (CM), and 38 with attackers (AT) players. In this study, we did not aim to determine the contextual influences (e.g., home vs. away matches) on top-up practices since a previous study had already addressed this issue [20]. For this reason, and to increase the statistical power of the analyses, the data from the observations were pooled per playing position.

### Participants

Twenty-six elite-level outfield male soccer players (mean ± SD; age: 25.4 ± 4.2 years, body mass: 77.7 ± 5.9 kg, height: 182.5 ± 6.8 cm, and body fat: 9.1 ± 1.1%) from a team in Portugal’s first league participated in this study. Six of the players were recruited to play for their respective national teams during the study. The data were collected as part of the professional duties, in which players are continually monitored throughout the season. The Ethics Committee of the University of Maia approved this study (n° 210/2024). The procedures followed the ethical principles outlined in the Declaration of Helsinki (2013), and all data were anonymized for the analysis to ensure players’ confidentiality.

### Procedures

#### Top-up sessions

The top-up training sessions for the non-called-up players were performed during the morning of match day (MD) and consisted of activation drills, followed by soccer passing drills, mini goal games, and finalization exercises with goalkeeper (1 × 1+GK; 2 × 1+GK). The post-match top-up sessions (MD+1) consisted of activation drills followed by rondos and 3 situations of 8 to 10 min (4 × 4; 5 × 5; 6 × 6) of SSG (35 × 40 m; 60 × 30 m) with goalkeepers. These were intercepted by 2 sets of 2 × 5–10 seconds high straight-line running intervals performed between the halfway line and the goal line. Due to the marked differences between the top-ups of the non-called-up players and the post-match top-ups, only the latter were considered for analysis to reduce the variability of the measures. It is important to mention that the soccer content of these sessions (i.e., MD+1) was highly typical, but the external load could vary slightly based on the number of players, contextual influences (away vs home matches), and the objective of each session (congested or not congested phases).

### External load assessment

The activity profile of the players was monitored in each training session using a portable 10 Hz global positioning system (GPS) unit (Vector S7, Catapult Sports, Melbourne, Australia). Each player wore the same GPS unit during each observation in order to disregard inter-unit errors [26]. The units were placed in a specially designed vest, inside a mini pocket positioned between the shoulder blades. The coefficient of variation (CV%) of GPS units used during intermittent exercise has previously been reported as 1.6–5% for a range of running and speed measures [27]. The data were extracted from each unit and split using the unit software (Catapult Innovations).

Global Positioning Systems-derived and accelerometer-derived variables relating to the total distance (TD), total player load, running distance (14.4 to 19.7 km · h^−1^), high-speed running (HSR; 19.8 to 25.1 km · h^−1^), and sprint distances (≥ 5.2 km · h^−1^), frequency of sprints, and medium and intense accelerations/decelerations (ACC/DEC; 2–3 m · s^−2^ and > 3 m · s^−2^) were quantified. The fixed speed thresholds used have been established based on previous studies [28]. All variables were calculated in absolute and relative terms per minute of play.

### Statistical analysis

A post-hoc power analysis was conducted using the GLIMMPSE 3.1.3 online calculator for general linear mixed model power (available at: https://glimmpse.samplesizeshop.org). The analysis determined a statistical power of 1.0. The Hotelling Lawley Trace test was chosen, with the alpha level set at 0.05. A minimum sample size of 38 observations was specified in the analysis. High-speed running was included as the dependent variable, while the condition (top-up and matches) was modeled as the time effect. A linear mixed-effects model with restricted maximum likelihood estimations was used to examine differences within playing positions between matches and top-up conditioning sessions. Mixed models can account for unbalanced repeats per player and thus can be used to model the data. For count variables (number of accelerations, number of decelerations, number of sprints) a generalized mixed model using Poisson distribution was used. Playing position and their interaction were modeled as fixed effects (effects describing the association between the dependent variable and covariates), while ‘athlete ID’ was included as a random effect (effects generally representing random deviations from the relationships of the fixed part of the model). Post-hoc pairwise comparisons between the estimated marginal means were performed. An α-level of 0.05 was used as the level of significance for statistical comparisons. The effect size (Cohen’s d ES) was calculated from the ratio of the mean difference to the pooled standard deviation and interpreted as follows: trivial < 0.2; 0.2 ≤ small < 0.6; 0.6 ≤ moderate < 1.2; 1.2 ≤ large < 2.0; very large > 2.0 [29]. All statistical analyses were conducted using the Jamovi statistical package (2020, version 1.6).

## RESULTS

The top-up session duration was lower (54.61 ± 8.37 minutes) than that of soccer matches (84.30 ± 18.61 minutes), regardless of the playing position (all p < 0.001).

## Running demands during matches and post-match top-up sessions

**FIG. 1:**
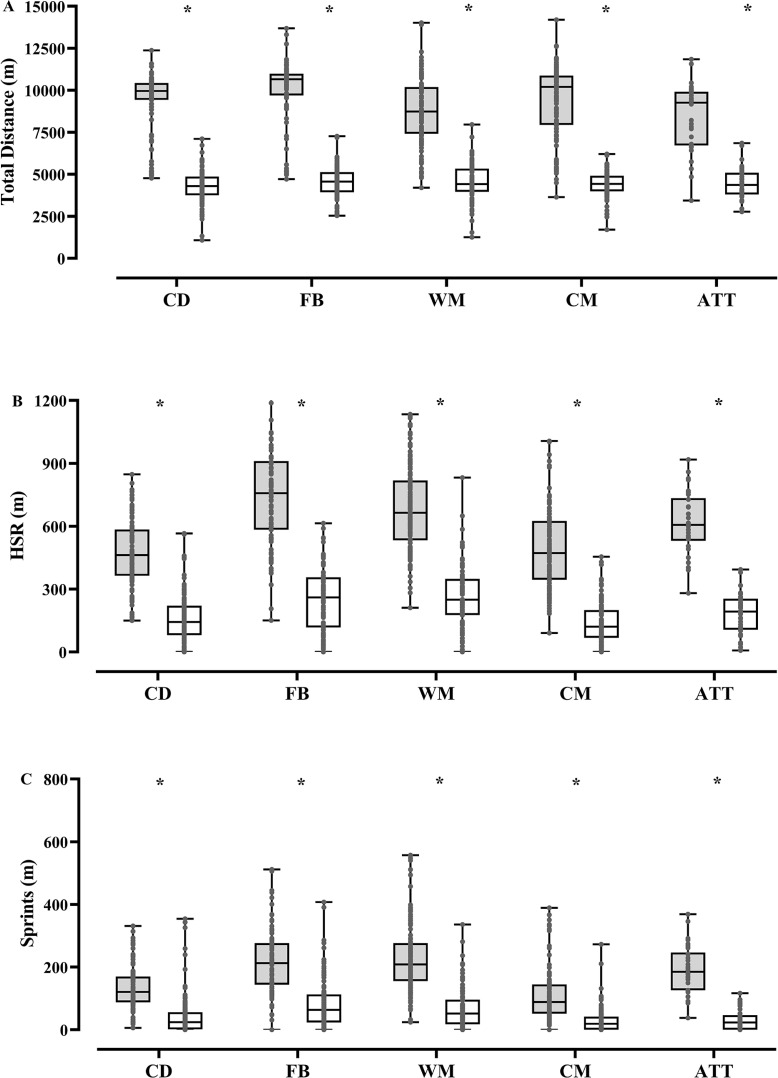
Box-plots of absolute running demands during matches (dark box) and top-ups sessions (light box) in soccer players according to playing positions. HSR = high-speed running; * = statistical difference between matches and top-ups sessions (p < 0.05).

There were statistical differences in running demands between soccer matches and post-match top-up sessions (Table 1 and Figure 1; all p-values < 0.001). Irrespective of playing position, running demands were greater during soccer matches than those in top-up sessions. Very large ES were found for TD (ES range: 2.46 to 3.74; p < 0.001), running (ES range: 2.93 to 3.44; p < 0.001), HSR (ES range: 2.21 to 3.47; p < 0.001), and player load (ES range: 2.34 to 3.23; p < 0.001) between soccer matches and post-match top-up sessions. In addition, moderate to very large ES were observed for sprinting distance (ES range: 1.00 to 2.76; p < 0.001) between soccer matches and post-match top-up sessions.

**TABLE 1:** Absolute running demands during matches and post-match top-ups sessions for elite soccer players according to playing positions.

Variables	Position	Matches	Top-ups	Cohen’s *d*	IC95%	Difference	SE	t	p-value
Total Distance (m)	CD	9327.9 ± 1821.6	4306.2 ± 975.1	3.5	3.0 to 3.9	5092.8	22.0	22.8	< 0.001
	FB	10007.3 ± 1922.0	4540.8 ± 884.7	3.7	3.2 to 4.2	5502.9	29.6	18.6	< 0.001
	WM	8713.5 ± 1973.9	4522.8 ± 1143.1	2.4	2.0 to 2.8	4133.3	25.1	16.5	< 0.001
	CM	9330.1 ± 2118.1	4401.6 ± 781.7	3.0	2.6 to 3.4	4920.5	22.4	21.9	< 0.001
	ATT	8394.4 ± 2049.5	4462.1 ± 910.8	2.5	1.9 to 3.2	3419.9	46.5	7.4	< 0.001

Running (m)	CD	1289.5 ± 288.7	360.8 ± 182.8	3.9	3.4 to 4.3	908.2	38.5	23.6	< 0.001
	FB	1563.9 ± 394.2	447.9 ± 192.4	3.6	3.2 to 4.1	1097.4	52.2	21.0	< 0.001
	WM	1276.1 ± 336.9	432.9 ± 181.2	2.9	2.5 to 3.3	850.1	43.5	19.5	< 0.001
	CM	1502.1 ± 414.5	363.9 ± 134.8	3.6	3.1 to 4.0	1132.4	39.4	28.7	< 0.001
	ATT	1129.6 ± 317.6	409.6 ± 151.6	2.9	2.2 to 3.6	616.5	79.2	7.8	< 0.001

HSR (m)	CD	470.9 ± 160.7	161.1 ± 118.2	2.2	1.8 to 2.5	296.1	22.3	13.3	< 0.001
	FB	761.1 ± 258.1	250.7 ± 147.8	2.4	2.0 to 2.8	513.0	30.3	16.9	< 0.001
	WM	684.8 ± 199.8	245.6 ± 146.4	2.3	2.0 to 2.7	435.8	25.3	17.3	< 0.001
	CM	488.8 ± 189.6	140.2 ± 99.5	2.2	1.9 to 2.6	321.7	22.8	13.3	< 0.001
	ATT	614.6 ± 149.8	180.7 ± 98.5	3.4	2.7 to 4.2	433.9	48.7	8.9	< 0.001

Sprint (m)	CD	125.6 ± 69.1	44.3 ± 66.2	1.1	0.8 to 1.4	82.0	10.3	8.0	< 0.001
	FB	215.9 ± 103.7	74.1 ± 70.1	1.6	1.2 to 1.9	154.0	13.9	11.1	< 0.001
	WM	227.7 ± 107.7	59.1 ± 68.1	1.8	1.5 to 2.1	158.8	11.6	13.6	< 0.001
	CM	108.3 ± 85.1	38.4 ± 41.5	1.0	0.7 to 1.3	63.6	10.5	6.1	< 0.001
	ATT	193.1 ± 78.2	32.1 ± 33.6	2.7	2.1 to 3.4	161.1	22.4	7.2	< 0.001

Player load (a.u.)	CD	841.9 ± 170.5	448.4 ± 104.2	2.9	2.4 to 3.2	411.6	22.9	17.9	< 0.001
	FB	1147.2 ± 271.2	506.0 ± 98.8	3.2	2.7 to 3.6	609.5	31.2	19.5	< 0.001
	WM	907.2 ± 199.2	485.2 ± 129.3	2.4	2.0 to 2.7	423.9	25.6	16.6	< 0.001
	CM	888.4 ± 211.2	449.2 ± 89.3	2.6	2.2 to 3.0	423.5	23.5	18.1	< 0.001
	ATT	797.1 ± 180.5	463.9 ± 98.6	2.3	1.7 to 2.9	321.4	49.6	6.5	< 0.001

TD = total distance; HSR = high-speed running; Pl = player load; CD = central defender; FB = fullback; WM = wide midfielder; CM = central midfielder; ATT = attackers.

There were statistical differences in running demands between matches and top-up sessions in relative terms (per minute) (Table 2, all p-values < 0.001). Irrespective of playing position, running demands were greater during soccer matches than those in top-up sessions. Very large ES were found for TD (ES range: 2.27 to 3.00; p < 0.001) and running (ES range: 2.71 to 3.48; p < 0.001) between soccer matches and top-up sessions. In addition, players covered greater distances in HSR (ES range: 1.34 to 3.64; p < 0.001), sprint (ES range: 0.74 to 1.77; p < 0.001), and accumulated greater player load (ES range: 1.09 to 2.12; p < 0.001) during soccer matches compared to top-up sessions.

**TABLE 2:** Relative running demands during matches and post-match top-ups sessions for elite soccer players according to playing positions.

Variables	Position	Matches	Top-ups	Cohen’s *d*	IC95%	Difference	SE	t	p-value
Total Distance (m · min^-1^)	CD	104.5 ± 5.2	78.5 ± 12.5	2.6	2.2 to 2.9	26.3	1.5	17.0	< 0.001
	FB	110.1 ± 6.0	83.3 ± 14.6	2.3	1.9 to 2.6	29.2	1.9	14.6	< 0.001
	WM	112.0 ± 9.6	83.8 ± 15.0	2.3	1.9 to 2.6	32.9	1.7	19.7	< 0.001
	CM	112.8 ± 9.9	81.3 ± 10.9	3.0	2.6 to 3.4	31.5	1.5	20.8	< 0.001
	ATT	104.3 ± 9.2	80.2 ± 11.3	2.2	1.6 to 2.8	26.4	3.1	8.5	< 0.001

Running (m · min^-1^)	CD	14.5 ± 2.3	6.5 ± 2.9	2.9	2.5 to 3.3	7.7	0.4	19.1	< 0.001
	FB	17.1 ± 2.9	8.3 ± 3.5	3.2	2.8 to 3.6	9.1	0.5	16.8	< 0.001
	WM	16.4 ± 3.0	8.0 ± 3.1	2.7	2.3 to 3.0	9.5	0.4	20.9	< 0.001
	CM	18.3 ± 3.8	6.7 ± 2.5	3.4	3.0 to 3.9	11.7	0.4	28.6	< 0.001
	ATT	14.0 ± 2.3	7.3 ± 2.3	2.8	2.1 to 3.4	6.8	0.8	8.2	< 0.001

HSR (m · min^-1^)	CD	5.2 ± 1.5	2.9 ± 1.9	1.3	1.0 to 1.6	2.2	0.3	7.7	< 0.001
	FB	8.3 ± 2.4	1.5 ± 1.2	3.6	3.1 to 4.1	4.1	0.4	10.6	< 0.001
	WM	8.8 ± 1.9	4.7 ± 2.9	1.7	1.4 to 2.0	4.8	0.3	14.8	< 0.001
	CM	5.9 ± 1.9	2.6 ± 1.9	1.7	1.4 to 2.0	3.1	0.3	10.4	< 0.001
	ATT	7.8 ± 2.1	3.3 ± 1.9	2.2	1.6 to 2.8	5.1	0.6	8.4	< 0.001

Sprint (m · min^-1^)	CD	1.4 ± 0.7	0.7 ± 1.0	0.7	0.5 to 1.0	0.6	0.1	4.6	< 0.001
	FB	2.3 ± 1.0	1.5 ± 1.2	0.7	0.4 to 1.0	1.2	0.2	6.7	< 0.001
	WM	2.9 ± 1.2	1.2 ± 1.2	1.3	0.9 to 1.6	1.8	0.2	11.9	< 0.001
	CM	1.2 ± 0.8	0.5 ± 0.8	0.8	0.5 to 1.1	0.5	0.1	3.7	< 0.001
	ATT	2.5 ± 1.4	0.6 ± 0.6	1.7	1.21 to 2.34	2.1	0.3	6.9	< 0.001

Player load (a.u.)	CD	9.4 ± 0.8	8.1 ± 1.3	1.0	0.8 to 1.3	1.7	0.2	10.7	< 0.001
	FB	12.5 ± 1.5	9.2 ± 1.5	2.1	1.7 to 2.3	3.1	0.2	14.5	< 0.001
	WM	11.6 ± 1.0	8.9 ± 1.6	2.0	1.7 to 2.3	3.4	0.2	18.9	< 0.001
	CM	10.7 ± 1.4	8.3 ± 1.3	1.7	1.4 to 2.4	2.4	0.2	14.8	< 0.001
	ATT	9.9 ± 1.1	8.3 ± 1.2	1.3	0.8 to 1.9	2.3	0.3	7.3	< 0.001

TD = total distance; HSR = high-speed running; Pl = player load; CD = central defender; FB = fullback; WM = wide midfielder; CM = central midfielder; ATT = attackers.

## Frequency of running demands during matches and post-match top-up sessions

**FIG. 2:**
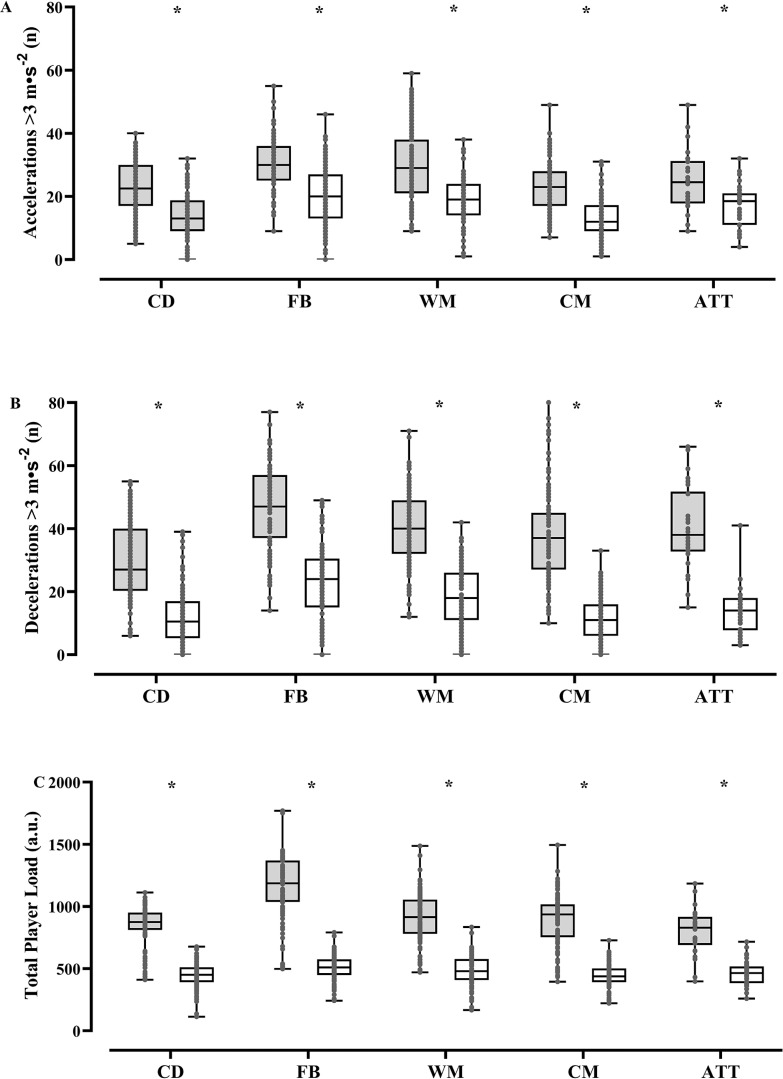
Box-plots of frequency of accelerations and decelerations (> 3 m·s-2) and player load during matches (dark box) and top-ups sessions (light box) in soccer players according to playing positions. * = statistical difference between matches and top-ups sessions (p < 0.05).

There were statistical differences in the frequency of running demands between soccer matches and top-up sessions (Figure 2; p < 0.05). Specifically, a greater number of accelerations, decelerations, and sprints were performed during soccer matches compared to the top-up sessions (p < 0.05). Large to very large ES were found for ACC 2–3 m · s^−2^ (ES range: 1.48 to 2.18; p < 0.001), DEC 2–3 m · s^−2^ (ES range: 1.93 to 3.93; p < 0.001), and DEC > 3 m · s^−2^ (ES range: 1.91 to 2.66; p < 0.001) between soccer matches and post-match top-up sessions. In addition, moderate to large ES were reported for ACC > 3 m · s^−2^ (ES range: 0.98 to 1.37; p < 0.005) and moderate to very large ES were found for the number of sprints (ES range: 1.00 to 2.44; p < 0.001) between soccer matches and top-up sessions.

There were statistical differences in the frequency of running demands between matches and top-up sessions in relative terms (per minute) (Table 3, p < 0.005). Irrespective of playing position, a greater number of DEC 2–3 m · s^−2^ (ES range: 0.27 to 1.74; p < 0.005), DEC > 3 m · s^−2^ (ES range: 0.50 to 2.21; p < 0.001), and sprints (ES range: 0.65 to 1.74; p < 0.005) were performed during soccer matches compared to the top-up sessions. However, the statistical differences were position-specific for the number of ACC 2–3 m · s^−2^, with no differences found for CD (p = 0.065; ES = 0.33) and ATT (p = 0.532; ES = 0.22). Regarding ACC > 3 m · s^−2^, no differences were reported for CD (p = 0.251; ES = 0.00), CM (p = 0.346; ES = 0.31), and ATT (p = 0.876; ES = 0.00) between soccer matches and top-up sessions.

**TABLE 3:** Relative frequency of running demands during matches and post-match top-ups sessions for elite soccer players according to playing positions.

Variables	Position	Matches	Top-ups	Cohen’s *d*	IC95%	Difference	SE	t	p-value
ACC 2–3 m · s^-2^ (n · min^-1^)	CD	1.19 ± 0.23	1.09 ± 0.35	0.3	0.1 to 0.6	0.07	0.04	1.84	0.065
	FB	1.39 ± 0.17	1.35 ± 0.43	0.1	-0.1 to 0.4	0.24	0.05	4.66	< 0.001
	WM	1.39 ± 0.25	1.25 ± 0.39	0.4	0.1 to 0.7	0.31	0.04	7.27	< 0.001
	CM	1.30 ± 0.16	1.10 ± 0.31	0.8	0.5 to 1.1	0.19	0.04	4.85	< 0.001
	ATT	1.19 ± 0.16	1.14 ± 0.27	0.2	-0.2 to 0.7	0.07	0.03	0.64	0.532

ACC > 3 m · s^-2^ (n · min^-1^)	CD	0.25 ± 0.07	0.25 ± 0.13	0.0	-0.2 to 0.3	-0.01	0.01	-1.47	0.251
	FB	0.33 ± 0.08	0.37 ± 0.17	-0.3	-0.5 to 0.0	0.06	0.02	2.93	0.003
	WM	0.39 ± 0.21	0.35 ± 0.14	0.2	-0.0 to 0.5	0.08	0.02	5.03	< 0.001
	CM	0.27 ± 0.07	0.24 ± 0.12	0.3	0.0 to 0.6	0.01	0.01	0.94	0.346
	ATT	0.30 ± 0.09	0.30 ± 0.11	0.0	-0.5 to 0.5	0.01	0.03	0.15	0.876

DEC 2–3 m · s^-2^ (n · min^-1^)	CD	1.07 ± 0.23	0.89 ± 0.36	0.5	0.3 to 0.9	0.16	0.04	4.18	< 0.001
	FB	1.42 ± 0.19	1.35 ± 0.30	0.2	-0.0 to 0.6	0.36	0.05	6.68	< 0.001
	WM	1.38 ± 0.21	1.11 ± 0.38	0.9	0.6 to 1.2	0.41	0.04	9.16	< 0.001
	CM	1.38 ± 0.18	0.91 ± 0.34	1.7	1.4 to 2.1	0.40	0.04	9.92	< 0.001
	ATT	1.32 ± 0.18	0.97 ± 0.24	1.6	1.0 to 2.1	0.34	0.08	4.01	0.002

DEC > 3 m · s^-2^ (n · min^-1^)	CD	0.32 ± 0.11	0.22 ± 0.15	0.7	0.4 to 1.0	0.08	0.01	5.12	< 0.001
	FB	0.51 ± 0.11	0.42 ± 0.22	0.5	0.2 to 0.8	0.22	0.02	9.44	< 0.001
	WM	0.52 ± 0.12	0.33 ± 0.17	1.3	1.0 to 1.6	0.27	0.02	13.64	< 0.001
	CM	0.45 ± 0.13	0.21 ± 0.12	1.9	1.6 to 2.2	0.20	0.02	11.01	< 0.001
	ATT	0.49 ± 0.10	0.24 ± 0.12	2.2	1.6 to 2.8	0.25	0.03	6.81	< 0.001

Number of sprints (n · min^-1^)	CD	0.05 ± 0.03	0.02 ± 0.03	1.0	0.7 to 1.3	0.03	0.01	4.86	< 0.001
	FB	0.08 ± 0.04	0.05 ± 0.05	0.6	0.4 to 0.9	0.04	0.01	5.47	< 0.001
	WM	0.11 ± 0.04	0.04 ± 0.04	1.7	1.4 to 2.1	0.07	0.01	11.74	< 0.001
	CM	0.04 ± 0.03	0.02 ± 0.03	0.6	0.4 to 0.9	0.02	0.01	3.12	0.002
	ATT	0.08 ± 0.05	0.02 ± 0.03	1.4	0.9 to 2.0	0.06	0.01	5.43	< 0.001

ACC = accelerations; DEC = decelerations; CD = central defender; FB = fullback; WM = wide midfielder; CM – central midfielder; ATT = attackers.

## DISCUSSION

In this study, we compared accumulated running demands during competitive matches and post-match top-up sessions in non-starters considering the playing position of the players. Our main findings revealed that, regardless of playing position, non-starters accumulated significantly lower loads during top-up sessions compared to those accumulated during the matches for most of the variables analyzed. On average, running demands during top-up sessions did not exceed 40% of match-play load, particularly for HSR (range: 28.7 to 35.8%) and sprinting (range: 16.6 to 35.7%) distances, and number of sprints (range: 18.8 to 38.9%). Therefore, soccer coaches should consider these findings while planning top-up sessions for non-starters (< 45 min of match participation) in order to replicate the high-intensity demands of competition and maintain squad’s physical fitness and readiness to play.

Top-up or compensatory training sessions are commonly prescribed for non-starters to compensate for their insufficient match load [20, 21], playing a crucial role in maintaining fitness levels and match readiness in these players who accumulate significantly lower match demands [30]. Nonetheless, these sessions typically result in lower TD and distances covered at different locomotor categories compared to actual matches (ES range: 1.00 to 3.90; large to very large), regardless of playing position (Table 1). Findings from Marcin et al. [31] reinforce that, although non-starters demonstrate higher activity levels during the final stages of matches, their cumulative sprint and HSR distances remain significantly lower than those of starters when compared across playing positions and full match durations. Of note, non-starters also have ~30% less training time during top-up sessions than players who completed > 45 min in a match, which may partially contribute to these differences. Nevertheless, players also covered greater distances, in relative terms, during soccer matches in comparison to top-up sessions (ES range: 0.74 to 3.64; moderate to very large). Previous data from European soccer clubs showed that starters accumulated higher workloads than nonstarters over a typical in-season period, primarily due to match time [3, 8, 21, 32]. This is particularly relevant for coaching staff since accumulating insufficient playing time may affect the team’ physical fitness over the season [13, 14]. In fact, it was demonstrated that players who accumulated more match time experienced greater improvements in muscle strength and sport-related performance (i.e., sprints, jumps, and change of direction) [14]. Therefore, soccer coaches are recommended to incorporate extra top-up sessions for non-starters during the week to mitigate undesirable detraining effects during the competitive season.

Regarding HSR and sprint events, compensatory training sessions only account for ~20–35% of the workload compared to matches (ES range: 2.21 to 3.47 and 1.00 to 2.76, respectively). Previous data from an elite soccer club showed that non-starters accumulated significantly lower HSR and sprinting distances (20–30%) during top-up sessions using SSG drills (30–60 m^2^ per player) [21], indicating their inability to effectively replicate match intensities. In practice, the increased hamstring muscle injury risk related to spikes in sprint loads [11, 33] may limit coaches from overloading nonstarters during top-up sessions. Despite the complex nature of the workload-injury relationship, it appears that under-loading non-starters for sprint runs may also increase their risk of injury [15, 16]. Adequate exposure to HSR and sprint events could represent a “winwin” strategy for the management of soccer-specific fitness and injury mitigation [17, 34], especially for those players who are usually listed as non-starters. It has been proposed that soccer players should accumulate a weekly workload of ~2 × match loads to maintain adequate sprint stimuli and their readiness to play [3, 22, 35]. In practice, soccer coaches could schedule extra top-up sessions on the MD-4 and MD-3 for non-starters to compensate for their lack of match-play load. Due to the uncertainty about upcoming match selection, this specific time window could be used to overload nonstarters, ensuring their availability for selection (i.e., reducing residual fatigue) before matches. Further research is needed to validate this approach, but it presents a promising strategy for optimizing player readiness and performance.

The lower values found for HSR and sprint events during top-up sessions compared to matches can be attributed to the SSG drills usually prescribed by the coaching staff. The restricted space in SSG drills (< 100 m^2^ per player) may limit players from achieving and sustaining near-maximal sprints [36, 37]. Previous research has demonstrated that a minimum area per player of ~200–300 m^2^ in SSG is necessary to replicate the match demands for HSR and sprint runs in elite soccer players [38]. Thus, large-sided games or friendly matches are often considered ideal for simulating match scenarios, as they provide a more accurate representation of the physical demands players experience during actual competitions. These formats allow for higher HSR and sprinting volume. However, the practical implementation of these training methods can be challenging due to logistical constraints, such as tight match schedules or the need for additional resources, like opposing teams. As a result, while these training methods are highly effective, they may not always be feasible in a regular training cycle. To address this limitation, coaching staff are encouraged to creatively integrate SSG drills with running-based drills to provide sufficient stimuli for HSR and sprint runs during top-up sessions, as these formats can enhance both of these metrics [39]. Future studies should examine top-up sessions using SSG with a large relative area per player (≥ 200 m^2^) and/or incorporate other training modes (i.e., running-based drills) for non-starters to overload near-maximum sprints. However, it is difficult to program such large SSG due to the reduced number of players typically involved in the compensatory training sessions, as starters or players who accumulate > 45 minutes in the previous matches are usually involved in recovery training sessions.

In the present study, we also compared the frequency of ACC and DEC at different intensities during top-up sessions compared to soccer matches. Significant differences were found in the number of accelerations, decelerations, and sprints between top-ups sessions and soccer matches, regardless of playing position (ES range: 0.98 to 3.93). On average, the SSG drills used during top-up sessions exceeded 50% of match-play demands for ACC 2–3 m · s^−2^ (~60%) and ACC > 3 m · s^−2^ (~65%). Comparing our findings with previous studies should be done with caution due to variations in acceleration thresholds and differences in tracking systems used to measure acceleration variables in team sports [25]. Nevertheless, our results are consistent with data from an elite Dutch team, indicating that compensatory training at MD+1 accounts for ~50% of medium (1.5–3.0 m · s^−2^) and ~60% of high (> 3 m · s^−2^) ACC demands from matches [3]. In contrast, previous data from an elite Spanish team showed that top-up sessions exceeded 80% of matchplay ACC (> 3 m · s^−2^) demands [21]. Explaining these discrepancies among studies is difficult; however, the training approaches (i.e., SSG formats) implemented by different soccer clubs might substantially account for this workload variability across various soccer leagues [40]. For this reason, future studies should explore different game formats during the top-up sessions to promote variable training stimuli across the locomotor activities required in soccer.

Top-up sessions usually encompassed SSG drills in relatively smaller areas than those used in official matches [3, 19, 21]. Therefore, non-starters were able to compensate for the number of ACC (~60%) to a greater extent than HSR and sprint events (~20–35%). Of note, no differences were found, in relative terms, for CD and ATT in the number of ACC 2–3 m · s^−2^ and for CD, CM, and ATT in the number of ACC > 3 m · s^−2^ between soccer matches and top-up sessions. On the other hand, the number of DEC at 2–3 m · s^−2^ (~50%) and > 3 m · s^−2^ (~40%) on average did not exceed 50% of matchplay loads. This is particularly relevant for scheduling weekly training practices for non-starters due to the association of DEC with muscle damage and fatigue (i.e., large eccentric loading) and their contribution to maneuvers such as changes of direction [25, 41], which are relevant in soccer [42]. During a regular in-season microcycle, both starters and non-starters accumulated ACC and DEC demands amounting ~3 times the match-loads [3, 19, 21], which is in line with previous recommendations [35]. Moreover, the difference in ACC and DEC between starters and non-starters is lower compared to other metrics such as HSR and sprints [3], suggesting that compensating for these high-intensity runs represents the main objective of top-up sessions [20]. These sessions could be strategically scheduled across microcycles using the concept of distributed practice or training microdoses [43].

There is a substantial amount of data available on position-specific physical loading in soccer matches [2, 5, 6]. For example, wide players tend to cover greater distances at high intensities than players in other positions to cope with the increased physical demands and tactical involvement (i.e., offensive and defensive) [5]. Therefore, it has been suggested that soccer coaches should consider a position-specific training strategy, including top-up sessions [19, 20, 22]. Our study revealed position-related differences in sprinting distances between top-up and soccer matches. For example, WM, FB, and ATT players exhibited large to very large ES (ES range: 1.62 to 2.76) in sprinting distances compared to CM and CD players (ES = 1.00 and 1.19, respectively). Previous studies have shown that wide players and attackers cover greater distances when sprinting than players in central positions [5, 6], making it more challenging for them to compensate during top-up sessions. Notably, SSG drills are not a “one-size-fits-all” training strategy to overload specific playing positions relative to match demands. This issue is compounded by the fact that positional roles can vary significantly across different teams and competitions, and tactical nuances further influence the physical demands placed on players in different positions [44]. Thus, our data highlight the importance of a position-specific training approach in top-up sessions to address variations in physical performance among different playing positions.

Moreover, compensatory training should also align with the team’s tactical philosophy. Coaches can enhance non-starters’ readiness by incorporating tactical elements such as quick transitions or high pressing into these sessions. For example, if the coach emphasizes fast transitions, compensatory drills should focus on rapid direction changes, acceleration, and high-speed coverage. By tailoring training to the tactical demands of the game, non-starters can better prepare for match-specific situations, improving both their physical fitness and tactical awareness [30].

The current study has limitations, such as relying only on external load metrics, which limit the analysis of players’ internal load responses. Additionally, the study does not account for contextual and tactical variables or key performance indicators, which are critical for a more comprehensive understanding of training and match demands. To improve future comparisons between matches and top-up sessions, incorporating metrics like perceived exertion and heart rate would be valuable. Additionally, monitoring running demands across the in-season microcycle could provide valuable insight into the weekly training structure for non-starters. From a practical perspective, the findings on top-up practices in an elite soccer club may still guide coaching staff in optimizing training for non-starters, there-by helping to balance physical fitness and match readiness. Future studies should examine both weekly external and internal loads of non-starters, particularly in elite players like those in this study. Moreover, future research should investigate individualized compensatory training protocols, considering factors such as playing position, match intensity, and player-specific needs. Additionally, future studies should also explore the long-term effects of compensatory training on physical fitness, performance adaptations, and injury resilience across different soccer teams and levels.

## CONCLUSIONS

The current study found that running demands during top-up sessions averaged less than 40% of match-play load. Non-starters, regardless of position, covered only 20–35% of the match distance in HSR and sprinting during MD+1 compensatory training. However, top-up sessions compensated more effectively for acceleration demands (~60%) than for high-speed running, particularly for certain positions (CD, CM, and ATT). Positions covering more sprinting distance during matches (wide and attacker players) faced greater challenges in compensating for the load during top-up sessions. To address the training load deficit, technical teams could implement large-sided games or running-based drills with increased relative areas per player to better replicate match demands. Scheduling additional top-up sessions on MD-4 and MD-3 could help ensure sufficient overload while maintaining readiness. Incorporating position-specific and tactical elements, such as high-intensity transitions or pressing drills, would further align training with match requirements.
